# Factors Predicting Blood Culture Positivity in Children With Enteric Fever

**DOI:** 10.1093/infdis/jiab357

**Published:** 2021-11-23

**Authors:** Manikandan Srinivasan, Kulandaipalayam Natarajan Sindhu, Karthikeyan Ramanujam, Ranjith Kumar Ramasamy, Sathyapriya Subramaniam, Santhosh Kumar Ganesan, Swathi Vajja, Anita Shirley David, Pramitha Lankala, Winsley Rose, Prabhakar D Moses, Nicholas C Grassly, Gagandeep Kang, Jacob John

**Affiliations:** 1 Division of Gastrointestinal Sciences, Wellcome Trust Research Laboratory, Christian Medical College, Vellore, India; 2 Department of Community Health, Christian Medical College, Vellore, India; 3 Wellcome Trust Research Laboratory, Division of Gastrointestinal Sciences, Christian Medical College, Vellore, India; 4 Department of Infectious Disease Epidemiology, Imperial College London, London, United Kingdom

**Keywords:** blood culture, children, enteric fever, India, *Salmonella* Typhi

## Abstract

**Background:**

Blood culture, despite low sensitivity, is the gold standard for enteric fever diagnosis. Understanding predictors of blood culture positivity may help design strategies to optimize enteric fever diagnosis.

**Methods:**

A cohort of 6760 children aged 0.5–15 years was followed for 3 years for enteric fever with blood cultures in an automated system, for fevers >3 days. Factors affecting test positivity in fevers and participant-level predictors for culture refusals were analyzed using regression models.

**Results:**

Overall, 6097 suspected typhoid/paratyphoid fever (STF) episodes were reported, of which 5703 (93.5%) STFs had sampling for blood cultures, with 394 (6.5%) refusals. *Salmonella enterica* serovar Typhi/Paratyphi positivity was culture-confirmed in 3.8% (218/5703) of STF episodes. Older children (odds ratio [OR], 1.96 [95% CI, 1.39–2.77]), larger blood volume inoculated (OR, 2.82 [95% CI, 1.71–4.66]), higher temperatures during fever (OR, 3.77 [95% CI, 2.89–4.91]), and fevers diagnosed as suspected typhoid or acute undifferentiated fever (OR, 6.06 [95% CI, 3.11–11.78]) had a higher probability of culture positivity. Antibiotics before culture did not decrease culture positivity. Blood culture refusals were higher for children from wealthier households or with milder illness.

**Conclusions:**

Performing blood cultures in older children with fever, especially those fevers with toxic presentation and increasing blood volume for inoculation are strategies to improve enteric fever detection in surveillance settings.

The global burden of typhoid and paratyphoid fever was estimated to be 14.3 million cases with 135 900 deaths in 2017 [1]. South Asia accounted for approximately 72% of global enteric fever cases, with a substantial burden in India [1]. The pooled incidence of typhoid fever in India was 377 per 100 000 population, with children being at the highest risk [2]. Given the substantial burden, the World Health Organization (WHO) has recommended the use of typhoid vaccines in endemic settings [3].

The introduction of typhoid vaccines in India requires an accurate estimate of enteric fever burden based on blood culture since blood culture is the gold standard diagnostic for typhoid/paratyphoid fever [4, 5]. However, blood culture has a low sensitivity of 40%–60%, requires special equipment and care to prevent contamination, and is not available everywhere [6]. On the other hand, the Widal test, which is widely available at low cost in India, has high false positivity, resulting in overdiagnosis and inappropriate antibiotic prescriptions [7–9]. Thus, in the absence of alternative standardized and validated tests, blood culture remains crucial in the accurate estimation of typhoid disease burden in low- and middle-income settings [5].

Studies based on blood culture have shown that duration of illness at the time of culture, age of the patient, prior use of antibiotics, and inoculating higher blood volumes in the culture bottle are associated with higher typhoid culture positivity [6, 10]. Prospective population-based surveillance of acute febrile illness additionally allows investigation of clinical and demographic characteristics that affect health care seeking and blood culture testing, as well as blood culture positivity. Identifying the participant-level characteristics associated with culture positivity could potentially allow the comparative application of these findings to those participants who presented with fever in the surveillance but refused a blood culture. This information is crucial in surveillance studies in developing settings to compute an accurate estimate of typhoid disease burden, by factoring in the underestimation, if any, that could have resulted due to the missing blood cultures from those subjects who were otherwise eligible to receive a blood culture for suspected typhoid fever. Furthermore, studying the clinical characteristics that predict blood culture positivity could help clinicians in urban settings to prioritize patients with febrile presentations to receive a blood culture, particularly during the early phase of illness to optimize the yield from blood culture, especially in those settings with limited resources.

Taking the opportunity of Surveillance for Enteric Fever in India (SEFI), a multisite prospective pediatric cohort for fever surveillance and enteric fever, we identified factors associated with blood culture positivity for enteric fever in children and, furthermore, the clinical and demographic factors associated with blood culture refusals.

## METHODS

During 2016–2017, a prospective pediatric cohort of approximately 6000 children, aged between 0.5 and 15 years, was recruited and followed up from 4 contiguous urban settlements in Vellore (Kaspa; Chinnallapuram [CAP]; Ramnaickanpalayam [RNP]; Vasanthapuram [VSPM]) for fever. This cohort was later nested within the SEFI, a multisite surveillance between 2017 and 2019 to estimate the incidence of blood culture–confirmed enteric fever in India [11]. During the surveillance, families of the study children were contacted weekly by designated field research assistants (FRAs) for identification of fevers. Fever in a child was defined as a parent/primary caregiver–reported rise in body temperature or a documented temperature >37.2°C (99°F) (a thermometer and a fever diary was given to the parent/primary caregiver, who was taught to document temperature during a fever). All fevers were followed up by field research assistants daily to document the temperature recorded during the fever episode, with details on visits and treatments sought at various health care facilities along with antibiotic usage until the resolution of the fever episode. Children with fevers for ≥3 days who met the study protocol criteria of “suspected typhoid/paratyphoid fever” (STF) were requested to visit the study clinic for clinical evaluation and a blood culture. Children with STF were evaluated by the study physician, in the majority of the cases, on day 4 of fever. The study physician ascertained the calendar days of fever and documented the clinical symptoms and antibiotics used, if any, during the fever episode. Children with STF satisfying the blood culture eligibility criteria (febrile for >12 hours before the clinic visit) were requested for a blood culture. If a culture was deferred for an STF when the child was afebrile in the last 12 hours, the child was followed up for the next 24 hours, with a blood culture being performed if there was a subsequent fever spike. Furthermore, the study physician made a provisional diagnosis during the clinical assessment, documenting the reason for not having performed a blood culture in cases where the culture was deferred or refused by the parent/primary caregiver. For children with STF who did not visit the clinic by day 4 or refused a blood culture, a home visit was made by the study supervisor documenting details of the fever episode, treatment sought, and the reason for not visiting the study clinic. Children with STFs found eligible for culture during home visits but who had not yet visited the study clinic were requested to visit the clinic, encouraging the families to continue treatment with the physician of their choice. All STFs were followed up until 3 fever-free days from the last day of defervescence, the third afebrile day marking the resolution of the fever episode. Following this, a final diagnosis was made by the physician based on clinical findings, investigations, and treatment received.

Blood sample for blood culture was collected and processed in BacT/ALERT automated system. Using age-based criteria, a blood volume of at least 3 mL and 5 mL was drawn for children aged ≤3 years and >3 to 15 years, respectively. The accurate volume of blood inoculated was determined by comparing the weight of the culture bottle measured pre- and post-inoculation of the sampled blood. Blood culture samples were transported within 4 hours of collection in ambient temperature to the Department of Clinical Microbiology, Christian Medical College, Vellore.

### Statistical Analysis

The baseline characteristics along with the categorical outcomes such as the number of eligible STFs, STFs that were blood culture positive for *Salmonella enterica* serovars Typhi/Paratyphi, and refusals for blood culture were expressed as percentages. Categorical variables such as clinical diagnosis, antibiotics used before blood culture, day of fever, outpatient visits, and seasonality were presented as percentages. Continuous variables such as age, fever duration (in days), body temperature (°C), and blood volume (mL) were expressed as median (interquartile range). The precise blood volume (mL) inoculated into the culture bottle was calculated using the formula:


$$\mathrm{Innoculated}\;\mathrm{blood}\;\mathrm{volume}\;(\mathrm{mL})=\frac{\text{Post weight}\left(\text{g}\right)-\text{Pre weight}(\text{g})}{1.06}$$

**Figure 1.**
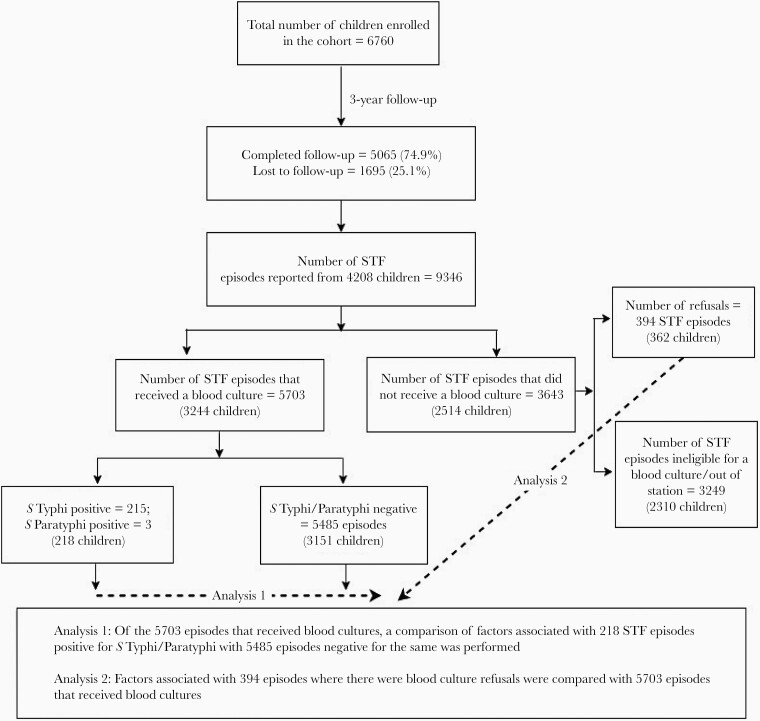
Flowchart depicting the number of children enrolled in the Vellore Surveillance for Enteric Fever in India cohort who were followed up for suspected typhoid fever (STF) with blood cultures being performed to detect *Salmonella enterica* serovars Typhi/Paratyphi among the eligible cases.

Broadly, this study undertook 2 analyses. First, to study the factors affecting blood culture positivity and the second being factors associated with culture refusals in the eligible STF episodes (Figure 1). Multilevel logistic regression was used for each of these analyses separately, considering the cluster effect at the level of subject and family, as appropriate. In the regression model studying factors affecting blood culture positivity, independent variables considered were age, day of fever when the blood sample was collected, blood volume, highest body temperature, initial clinical diagnosis and the month (to study the influence of seasonality) when the blood culture was performed. Also, an interaction term between the blood volume and age of the child was included in this model. To study factors associated with refusals for culture, predictors included in the model were sociodemographic characteristics of study children/family, characteristics of the febrile episode, treatment-seeking behavior, and clinical diagnosis of the fever episode. A *P* value < .05 was considered as the level of statistical significance. All statistical analyses were carried out using Stata version 14 software (StataCorp, College Station, Texas). Using the general additive models, nonlinear relationships between blood culture positivity with that of the age of the child was derived using cubic spline penalized regression in R software version 3.5.1 [12]. SEFI was approved by the Institutional Review Board (IRB) of Christian Medical College, Vellore (IRB Min number 10393).

## RESULTS

Of the 6760 children in the study cohort, 5073 (75%) were aged between 6 months and 10 years, with 4578 (69.9%) children belonging to the lower socioeconomic strata (Table 1). Comparison of demographic characteristics across the 4 study areas showed that VSPM had the highest proportion of children belonging to low socioeconomic status (80%) compared with CAP (72%), Kaspa (68%), and RNP (65%). Children from Kaspa had better access to an improved source of drinking water (Kaspa, 16%; RNP, 8%; CAP, 7%; and VSPM, 7%) and those from RNP had better access to sanitary facilities (RNP, 54%; CAP, 31%; VSPM, 31%; and Kaspa, 28%), compared to other areas. VSPM had the highest proportion of study households that practiced open-air defecation (35%) compared with other areas (CAP, 5%; RNP, 2.2%; and Kaspa, 0.3%) (data not shown).

**Table 1.** Sociodemographic Profile of the Children Enrolled in the Surveillance for Enteric Fever in India (SEFI) Cohort, Vellore, India (N = 6760)

Variable	No. (%)
Age (at enrollment)	
0.5–<5 y	2610 (38.6)
5–<10 y	2463 (36.4)
10–15 y	1687 (25)
Sex	
Male	3434 (50.8)
Female	3326 (49.2)
Religion^a^ (n = 6737)	
Hindu	3086 (45.8)
Muslim	3284 (48.8)
Christian	332 (4.9)
Other	35 (0.5)
SES^a^^,^^b^ (n = 6548)	
Low	4578 (69.9)
Middle	1777 (27.1)
High	193 (3)
Mother’s education^a^ (n = 6663)	
No formal education	943 (14.2)
Primary	1314 (19.7)
Middle	1646 (24.7)
Secondary	2311 (34.7)
Graduate and above	449 (6.7)
Type of family^a^^,^^c^ (n = 6548)	
Nuclear	3950 (60.3)
Joint/3-generation	2598 (39.7)
Type of house^a^^,^^d^ (n = 6548)	
Pucca	4789 (73.1)
Kutcha	172 (2.6)
Mixed	1510 (23.1)
Government house	77 (1.2)
Area of residence	
CAP	1778 (26.3)
Kaspa	2159 (31.9)
RNP	1795 (26.6)
VSPM	1028 (15.2)

Abbreviations: CAP, Chinnallapuram; RNP, Ramnaickanpalayam; SES, socioeconomic status; VSPM, Vasanthapuram.

^a^Missing data (religion, 23 subjects; SES, 212 subjects; mother’s education, 97 subjects; type of family, 212 subjects; and type of house, 212 subjects) as subjects dropped out/migrated out of study area before the demographic survey.

^b^SES classification was done using the modified Kuppusamy scale that accounted for occupation, education, and selected assets [25].

^c^Nuclear family is defined as a social unit comprising of parents living with their children; joint family as a family of siblings sharing a household along with their spouses and children; and 3-generation family as a family of son/daughter sharing a household with their parents, spouse, and children.

^d^Kutcha house is one constructed using mud, thatch, or other suboptimal-quality materials; pucca house is one constructed completely with optimal quality materials, including the floor, roof, and walls outside.

During the surveillance period of 3 years and 2 months, a total of 9346 STF episodes were reported from 4208 children. Of these 4208 children, 1821 (43.3%), 1072 (25.5%), and 1315 (31.3%) reported 1, 2, and 3 or more STF episodes, respectively, during the surveillance period. Overall, 6097 of the 9346 STF episodes reported were eligible for blood culture, of which 5703 (93.5%) episodes received the same. Blood culture refusals were recorded for 394 (6.5%) STF episodes (Figure 1). Blood culture positivity for *S* Typhi/*S* Paratyphi was 3.8% (218/5703 STF episodes), with 215 of 218 (98.6%) being *S* Typhi positive. In nearly 26% (1452/5703) of the STF episodes that resulted in blood cultures, the patient was on antibiotics prior to the culture. Respiratory infections (72.3%) and acute undifferentiated fevers (17%) were the most common provisional diagnosis documented for these STF episodes. Blood cultures for STFs were done predominantly on day 4 or 5 of fever (83% [4737/5703]; Table 2). The mean blood volume inoculated for cultures done in children between 1 and 3 years of age showed a steady rise from 1 to 3 mL, and this plateaued at 5 mL for 4 years and above, in concurrence with the protocol-specified requirements of blood volumes for culture (Supplementary Figure 1).

**Table 2.** Factors Associated With Salmonella Typhi/Paratyphi Blood Culture Positivity in the 5703 Suspected Typhoid Fever Episodes Reported From 3244 Surveillance for Enteric Fever in India (SEFI) Children at Vellore

Factor	STF Episodes Positive for *S* Typhi/Paratyphi (n = 218)	STF Episodes Negative for *S* Typhi/Paratyphi (n = 5485)	Univariate		Multivariate	
			OR (95% CI)	*P* Value	OR (95% CI)	*P* Value
Age at blood culture, y, median (IQR)	7.9 (5.7–10.7)	5.7 (3.5–9.1)	1.14 (1.09–1.19)	**<.001**	1.96 (1.39–2.77)	**<.001**
Age category at the time of blood culture						
0.5–<2 y	4 (1.8)	399 (7.3)	1	Ref		
2–<5 y	41 (18.8)	1965 (35.8)	2.12 (.72–6.24)	.173		
5–<10 y	102 (46.8)	2051 (37.4)	5.22 (1.80–15.09)	**.002**		
10–15 y	71 (32.6)	1070 (19.5)	7.47 (2.54–21.95)	**<.001**		
Sex						
Male	119 (54.6)	2911 (53.1)	1	Ref	…	
Female	99 (45.4)	2574 (46.9)	0.93 (.67–1.28)	.645	…	
Highest temperature during fever, °C, median (IQR)^a^	39.4 (38.7–39.8)	38.7 (38.1–39.2)	3.44 (2.69–4.40)	**<.001**	3.77 (2.89–4.91)	**<.001**
Initial clinical diagnosis^b^						
RTIs	99 (45.4)	3889 (71.0)	1	Ref	1	Ref
RTIs with other presentations^c^	9 (4.1)	128 (2.3)	3.20 (1.42–7.26)	**.005**	3.18 (1.29–7.87)	**.013**
Acute undifferentiated illness	67 (30.7)	901 (16.4)	3.43 (2.37–4.99)	**<.001**	2.96 (1.97–4.43)	**<.001**
Clinically suspected typhoid fever	26 (11.9)	153 (2.8)	7.73 (4.34–13.78)	**<.001**	6.06 (3.11–11.78)	**<.001**
Others	17 (7.8)	410 (7.5)	1.74 (.97–3.13)	.066	1.19 (.61–2.31)	.604
Antibiotic usage prior to blood culture						
Yes	55 (25.2)	1397 (25.5)	1.05 (.74–1.50)	.802	…	
No	163 (74.8)	4088 (74.5)	1	Ref	…	
Day of fever on which blood culture was performed						
1–3^d^	14 (6.4)	244 (4.4)	1.14 (.55–2.36)	.724	0.49 (.21–1.12)	.091
4	142 (65.1)	3334 (60.8)	0.90 (.58–1.39)	.635	0.73 (.44–1.20)	.218
5	29 (13.3)	1232 (22.5)	0.49 (.28–.86)	**.012**	0.48 (.26–.89)	**.021**
≥6	33 (15.1)	675 (12.3)	1	Ref	1	Ref
Blood volume inoculated for culture, mL^e^, median (IQR)	5.1 (4.8–5.3)	5 (4.7–5.2)	1.36 (1.15–1.60)	**<.001**	2.82 (1.71–4.66)	**<.001**
Seasonality						
Jan–Mar	19 (8.7)	1341 (24.5)	1	Ref	1	Ref
Apr–June	77 (35.3)	691 (12.6)	10.12 (5.67–18.06)	**<.001**	13.16 (6.89–25.16)	**<.001**
Jul–Sep	73 (33.5)	1442 (26.3)	3.85 (2.21–6.72)	**<.001**	4.23 (2.31–7.73)	**<.001**
Oct–Dec	49 (22.5)	2011 (36.7)	1.63 (.92–2.89)	.097	1.90 (1.03–3.52)	**.039**
Age × blood volume	…	…	…		0.90 (.84–.96)	**.001**

Data are presented as No. (%) unless otherwise indicated. Blood volume was added to the model as an interacting term with age of the child. The final model accounted for clustering at the family level. Intraclass correlation at family level was 0.39 and the variance was 2.11 (95% CI, 1.19–3.72). Values in bold represent significant *P* values (*P* < .05).

Abbreviations: CI, confidence interval; IQR, interquartile range; OR, odds ratio; RTI, respiratory tract infection; STF, suspected typhoid fever.

^a^Temperature was not recorded for 3 STF episodes.

^b^Initial diagnosis was not available for 4 STF episodes.

^c^Other presentations include acute gastroenteritis, urinary tract infections, acute hepatitis, or skin infections.

^d^Cultures were also performed when requested by the parent of the child, even for fevers <3 days.

^e^Information on blood volume was not available for 59 STF episodes.

**Figure 2.**
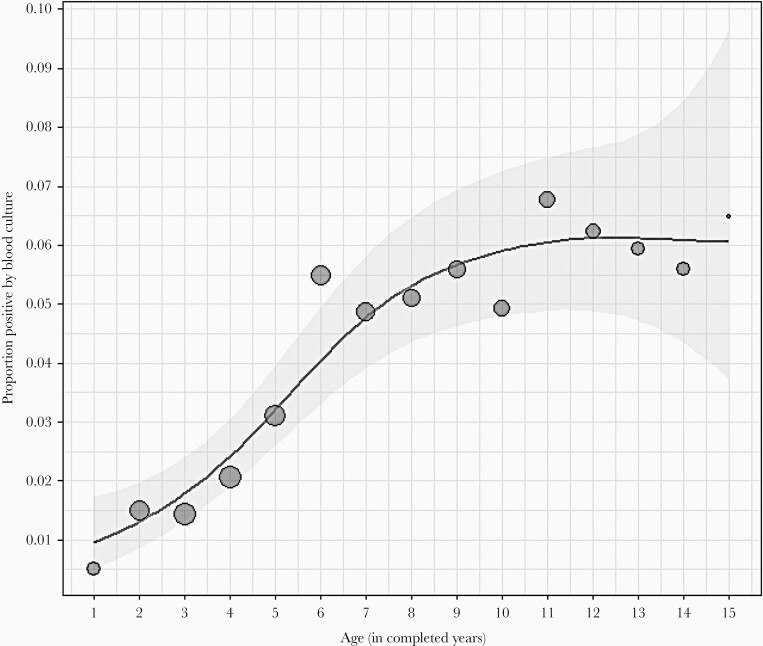
Relationship between blood culture positivity for *Salmonella enterica* serovars Typhi/Paratyphi and age of children in the Surveillance for Enteric Fever in India cohort. Position of each circle in the graph represents the proportion of blood cultures positive in children in each age group; size of circle represents the number of fever episodes included in each age category.

Among the 218 STF episodes positive for *S* Typhi/*S* Paratyphi, the most common provisional diagnosis assigned by the clinician was a respiratory infection in 108 (49.5%) episodes. In the multivariable model, significant factors associated with blood culture positivity were increasing age of the child (odds ratio [OR], 1.96 [95% confidence interval {CI}, 1.39–2.77]) and a higher blood volume inoculated for a culture (OR, 2.82 [95% CI, 1.71–4.66]), after adjusting for the clinical characteristics of children with STF episodes. This culture positivity further showed a linear relationship with the increasing age of the child up to 8 years of age, and this relationship plateaued thereafter (Figure 2). Clinical characteristics significantly associated with culture positivity were a higher peak temperature during the febrile episode (OR, 3.77 [95% CI, 2.89–4.91]) and those with an initial diagnosis of acute undifferentiated fevers (OR, 2.94 [95% CI, 1.94–4.46]) or STF (OR, 6.02 [95% CI, 3.07–11.80]). A seasonality was noted with blood culture positivity, with the probability of positivity being higher between April–June and July–September, which corresponds to the summer and monsoon seasons at Vellore, respectively (Table 2).

Factors associated with refusals for culture among children with STFs are presented in Table 3. In the multivariable analysis, children residing in VSPM, the study area with the highest proportion of children of low socioeconomic status, showed a lower probability of blood culture refusals (OR, 0.61 [95% CI, .37–.99]). Children with higher body temperature during the fever episode (OR, 0.58 [95% CI, .50–.68]) and prolonged duration of fever (OR, 0.55 [95% CI, .46–.66]) showed a lower probability of refusing a blood culture. Lower refusals were also noted in STFs subjectively perceived to be severe, lower respiratory infections (OR, 0.35 [95% CI, .20–.60]) and in those with more than a single diagnosis documented by the examining physician (OR, 0.21 [95% CI, .05–.94]). On the other hand, STFs that were classified as “others” that included fever with skin rash or mumps had a higher chance for blood culture refusals (OR, 4.12 [95% CI, 2.90–5.86]).

**Table 3.** **Factors Associated With Refusals for Blood Culture in the Eligible Suspected Typhoid Fever Episodes Reported in the Cohort (N = 6097)** ^a^

Factor	Refusals for Blood Culture (n = 394)^a^	STFs That Had a Blood Culture (n = 5703)^a^	Univariate		Multivariate	
			OR (95% CI)	*P* Value	OR (95% CI)	*P* Value
Age at the time of fever episode						
0.5–<2 y	31 (7.9)	407 (7.1)	1.42 (.85–2.37)	.176	…	
2–<5 y	143 (36.3)	2006 (35.2)	1.27 (.90–1.81)	.175	…	
5–<10 y	155 (39.3)	2153 (37.8)	1.31 (.93–1.84)	.125	…	
10–15 y	65 (16.5)	1137 (19.9)	1	Ref	…	
Sex						
Male	191 (48.5)	3030 (53.1)	0.84 (.66–1.06)	.146	…	
Female	203 (51.5)	2673 (46.9)	1	Ref	…	
Type of family^b^						
Nuclear	217 (55.8)	3176 (56.1)	0.98 (.76–1.28)	.904	…	
Joint/3-generation family	172 (44.2)	2490 (43.9)	1	Ref	…	
Religion^c^						
Hindu	145 (36.9)	2588 (45.4)	1	Ref	1	Ref
Muslim	228 (58.0)	2774 (48.7)	1.58 (1.21–2.06)	**.001**	1.36 (1.00–1.87)	.053
Christian	19 (4.8)	298 (5.2)	1.26 (.70–2.28)	.443	1.25 (.66–2.37)	.491
Others	1 (0.3)	37 (0.7)	0.45 (.05–4.29)	.486	0.58 (.06–5.81)	.645
Type of house^b^						
Pucca/mixed	383 (98.5)	5460 (96.4)	1	Ref	1	Ref
Kutcha/government	6 (1.5)	206 (3.6)	0.39 (.16–.98)	**.045**	0.52 (.20–1.36)	.183
Mother’s education^d^						
No formal education	39 (10.0)	554 (9.8)	1	Ref	…	
1–5 y	85 (21.7)	1056 (18.6)	1.35 (.82–2.21)	.229	…	
6–8 y	97 (24.7)	1534 (27.0)	0.96 (.59–1.55)	.877	…	
9–12 y	140 (35.7)	2061 (36.3)	1.03 (.65–1.63)	.889	…	
>13 y	31 (7.9)	473 (8.3)	1.05 (.57–1.92)	.874	…	
Presence of smartphone^b^						
Yes	307 (78.9)	4065 (71.7)	1.47 (1.09–1.99)	**.012**	1.34 (1.00–1.86)	.084
No	82 (21.1)	1601 (28.3)	1	Ref	1	Ref
Socioeconomic status						
Low	271 (69.7)	4010 (70.8)	1	Ref	…	
Middle	105 (27)	1495 (26.4)	1.01 (.78–1.35)	.941	…	
High	13 (3.3)	161 (2.8)	1.17 (.59–2.36)	.670	…	
Area						
Chinnallapuram	100 (25.4)	1350 (23.8)	1	Ref	1	Ref
Kaspa	115 (29.2)	1960 (34.3)	0.83 (.59–1.16)	.268	0.80 (.55–1.16)	.233
Ramnaickanpalayam	137 (34.8)	1477 (25.9)	1.35 (.96–1.81)	.081	1.18 (.81–1.73)	.395
Vasanthapuram	42 (10.7)	916 (16.0)	0.62 (.40–.96)	**.034**	0.61 (.37–.99)	**.046**
Antibiotic usage in first 3 days of fever						
Yes	109 (27.7)	1452 (25.5)	1.02 (.79–1.32)	.898	…	
No	285 (72.3)	4251 (74.5)	1	Ref	…	
Highest temperature during fever, °C, median (IQR)^e^	38.3 (37.8–39.0)	38.7 (38.1–39.2)	0.64 (.55–.73)	**<.001**	0.58 (.50–.68)	**<.001**
Duration of fever, d, median (IQR)	4 (4-4)	4 (4–5)	0.57 (.48–.67)	**<.001**	0.55 (.46–.66)	**<.001**
Final clinical diagnosis^f^						
Upper respiratory tract infection	203 (51.5)	3241 (56.9)	1	Ref	1	Ref
Acute febrile illness	81 (20.6)	967 (17.0)	1.35 (1.00–1.82)	.054	1.37 (1.00–1.88)	**.049**
Lower respiratory tract infection	17 (4.3)	746 (13.1)	0.33 (.19–.57)	**<.001**	0.35 (.20–.60)	**<.001**
Suspected typhoid fever	4 (1.0)	179 (3.1)	0.35 (.12–1.00)	**.050**	0.43 (.14–1.27)	.126
>1 of the above diagnoses	2 (0.5)	137 (2.4)	0.23 (.05–1.00)	**.047**	0.21 (.05–.94)	**.042**
Others^g^	87 (22.1)	425 (7.5)	4.06 (2.90–5.67)	**<.001**	4.12 (2.90–5.86)	**<.001**

The final model included random effects at both family and individual levels, with the intracluster correlation at family and individual level being 0.28 and 0.34, respectively. The variance at the level of family and individual subjects was 1.38 (95% CI, .83–2.31) and 0.29 (95% CI, .02–3.63), respectively. Values in bold represent *P* <.05.

Abbreviations: CI, confidence interval; IQR, interquartile range; OR, odds ratio; STF, suspected typhoid fever.

^a^Of the 6097 STF episodes, there was a total of 394 refusals for blood cultures among the 3606 children (the 3249 STFs that were finally not eligible for a blood culture were excluded) (Figure 1).

^b^Information on type of family, type of house, and smartphone was not available for 42 subjects.

^c^Information on religion was not available for 7 subjects.

^d^Information on mother’s education was not available for 7 subjects.

^e^Temperature recording was not available for 11 STF episodes.

^f^Final diagnosis was not available for 8 STF episodes.

^g^The top 3 causes associated with refusals were fever with rash (71.4%), mumps (30.4%), and viral fever (4.7%).

## Discussion

The Vellore SEFI cohort estimated an enteric fever culture positivity of 3.8% (95% CI, 3.3%–4.4%]) in children aged <15 years that showed a linear relationship with the increasing age of the child. Fever episodes with higher peak temperatures and those with a provisional physician diagnosis of suspected typhoid fever or acute undifferentiated fevers were more likely to be culture positive for typhoid. Children from lower socioeconomic strata, those with a prolonged fever episode, and those having higher temperatures were less likely to refuse a blood culture where there was a clinical suspicion of typhoid. Children with STFs who presented with fevers with perceived subjective severity were also less likely to refuse a blood culture.

Population-based studies done in South Asia and Africa have reported typhoid blood culture positivity rates between 2.6% and 6.4%, with those aged 5–9 years showing the highest positivity rate as observed in our study [13–16]. In our study, the rate of the proportion of typhoid positivity showed a steep rise from <1% to 5% in the age group 1–7 years, followed by a marginal increase of 5%–6% in the group aged 8–15 years (Figure 2). Systematic reviews have demonstrated blood volume to be an independent predictor for typhoid culture positivity, with the sensitivity of a blood culture test increasing by 3% for every additional milliliter of blood inoculated [12, 17]. This is in congruence with our study finding where the culture positivity increased with higher volumes of blood inoculated. Considering the required critical concentration of *S* Typhi in the blood of 1 colony-forming unit/mL to diagnose typhoid fever, it is plausible that the additional volume of blood inoculated added to the culture positivity yield [18]. Literature indicates that *S* Typhi blood culture yield could be improved with equivalence to that of a bone marrow culture with a 90% sensitivity when >10 mL of blood is inoculated [19]. However, blood draws of >10 mL, especially in children, are challenging in surveillance settings such as ours. Hence, a blood volume of approximately 7 mL is suggested, giving a 90% chance of isolating *S* Typhi in blood culture [10, 19].

A higher peak temperature during a fever episode in our cohort of children was associated with a higher probability of blood culture positivity. This is similar to findings from a study in Nepal as well as Middle Eastern settings where a greater chance for typhoid positivity was demonstrated among fevers with temperatures >38°C (100.4°F) [12, 20]. The plausible explanation for this would be the peak temperatures during the fever episode reflects high systemic bacteremia, perhaps augmenting the probability of culture positivity [19]. This emphasizes that that fevers with toxic presentation should raise the suspicion for typhoid by physicians in endemic settings and be investigated with a blood culture.

The use of antibiotics before a blood culture did not influence culture positivity in our study. This is in contrast to the finding from a recent systematic review of studies between 1937 and 2008. The review reported a decrease in blood culture sensitivity for enteric fever when antibiotics were used prior to culture [10]. This contrasting finding from our surveillance could be explained by the fact that the BACTEC blood culture system was used in our surveillance, where the resins incorporated in blood culture media could have potentially neutralized the antibiotics in the inoculated blood samples. This finding is supported by typhoid surveillance studies from the South Asian settings of Bangladesh, Nepal, and Pakistan [21].

STFs in our study with a provisional diagnosis of “suspected typhoid” stood a higher chance of culture positivity of 15%, which was twice the recent Nepal study estimate of 7% [12]. This could be due to the strict surveillance criteria used in our study where a fever of ≥3 days called in for a blood culture, compared to the Nepal study where fevers of ≥2 days received a blood culture [11, 12]. “Acute undifferentiated fever” (AUF) as the next common initial diagnosis had a typhoid positivity of 7% in our study compared to a previous hospital-based study from the same setting (Vellore) that showed a typhoid positivity of 3.7% [22]. Thus, AUF presentations are associated with a higher likelihood for culture positivity for typhoid and must be investigated in clinical practice using blood cultures, preferably by the first week of illness.

A seasonal pattern was observed with high typhoid blood culture positivity during the summer and monsoon seasons in Vellore corresponding to April–June and July–September, respectively. This probably mirrors the typhoid outbreaks in our study during the surveillance period of 3 years with an outbreak in year 1 being precipitated by flooding following monsoon rains and the other outbreak in year 3 by the high summer temperatures. This surge in typhoid cases during extreme heat conditions and following rains is explained and supported by evidence from a recent systematic review [23].

A low refusal rate for blood culture was noted in the Vellore SEFI cohort (6.5%) when compared to the Nepal study (30%) [12]. This difference in refusal rates between the 2 studies can be explained by the different definitions used to identify fevers eligible for blood culture in the 2 studies (SEFI definition of ≥3 days of fever eligible for a blood culture vs the Nepal study definition of fevers up to 2 days or a temperature >38°C (100.4°F) being eligible for a culture). This perhaps led to more conservative blood cultures being performed at Vellore with subsequent lower culture refusals (frequent blood draws in children implying blood culture refusals in the future) [13]. The clinical severity of fever in our study, in terms of duration of illness or having higher temperatures and an initial physician diagnosis of suspected typhoid or lower respiratory infection, was associated with lower refusals, as also noted in the Nepal study [12]. Lower refusals among children with fevers of clinical severity could be because of primary caregivers perceiving these fevers to be “serious enough,” making them more inclined toward accepting a blood culture. Another interesting finding from our study was the high refusal rate for blood cultures in presentations such as fever with rash and mumps, illnesses that this community associates with religious sentiments (a fever with rash generally being attributed to the “wrath of god”). Furthermore, no visit to any health care facility is made nor are health personnel permitted into their homes during these illnesses, using only home-based remedies for management. Considering the lower refusal rates for blood culture in SEFI and the fact that the fever episodes where blood culture was not performed despite being eligible (because of participant’s refusal) were low, it is unlikely that these missing cultures could have resulted in underestimation of typhoid burden in our study.

SEFI is one of the very few large population-based studies from the Indian setting that estimated blood culture–based typhoid disease burden, using a cohort surveillance approach. The strength of the study is the highly intensive daily follow-up of fever episodes in children that facilitated real-time data capture on fever-related clinical characteristics, minimizing the recall bias that undermines the usually done hospital-based studies. We followed a strict surveillance blood culture eligibility definition of fever for ≥3 days in our study, as per the WHO guidelines, which provided a vital window of opportunity to compare our findings to those generated from other surveillance settings [5]. The blood culture testing rate in our study was high, with 93.6% of all eligible STFs receiving a culture. This study has a few limitations. Although it is known that that typhoid culture positivity increases with inoculation of larger blood volumes, the inoculated volume in our study was limited to <6 mL factoring in the parents’/primary caregivers’ concerns over the excessive blood draws (published elsewhere) [10, 24]. Moreover, although our study noted relatively lower body temperatures and shorter duration of fevers in children who refused a culture compared to those who did not, the actual difference noted is very small to draw concrete conclusions in real-world settings. While antibiotic usage for fevers in this surveillance was based on the primary caregiver’s history, underreporting of antibiotic use in this cohort is a possibility, particularly when no physician prescriptions were available, with the caregiver being unable to differentiate an antibiotic from other prescribed medications. This could have been overcome by performing tests to detect antibiotic levels in the urine giving a robust estimate of antibiotic usage in children, but this was beyond the scope of this large surveillance cohort [21].

## Conclusions

The potential inclusion of the typhoid conjugate vaccine within the immunization schedule in many developing countries like India necessitates the availability of a precise estimate of the typhoid disease burden in these settings. Given the high endemicity of typhoid fever in these settings and the fact that typhoid fever presents frequently with symptoms suggestive of respiratory infections, it is important to investigate fever episodes lasting 3 or more days, with or without an indication of a systemic focus of infection, with a blood culture. In the road toward typhoid control in India, the need of the hour also lies in reliable and inexpensive rapid diagnostic tests for typhoid, considering the challenges with the upscaling of blood culture–based surveillance in resource-constrained settings. Furthermore, incorporating factors associated with blood culture positivity for typhoid fever such as age, the volume of blood inoculated for culture, and clinical severity, as well as the adjustment for eligible subjects who did not opt for a blood culture within the surveillance program, are pivotal in estimating the true typhoid disease burden estimate.

## Supplementary Data

Supplementary materials are available at *The Journal of Infectious Diseases* online. Consisting of data provided by the authors to benefit the reader, the posted materials are not copyedited and are the sole responsibility of the authors, so questions or comments should be addressed to the corresponding author.

**Supplementary Figure 1.** Relationship between blood volume and age of children in the SEFI cohort.

jiab357_suppl_Supplementary_Figure_1

## Notes

***Author contributions.*** J. J. and G. K. conceived the study, drafted the protocol, and provided critical revision of the final draft. M. S., K. N. S., and K. R. were involved in the conduct of the study, did statistical analyses, and wrote and revised the manuscript. R. K. R., S. S., and S. K. G. did the data management and assisted in statistical analyses. N. G. provided epidemiological expertise. S. V., A. S. D., P. L., W. R., and P. D. M. provided the clinical support for the Surveillance for Enteric Fever in India (SEFI) cohort at Vellore. All authors approved the final version of the manuscript; agree to be accountable for all aspects of the work in ensuring that questions related to the accuracy or integrity of any part of the work are appropriately investigated and resolved; and confirm that all persons designated as authors qualify for authorship and all those who qualify for authorship are listed.

***Acknowledgments.*** We thank the parents and the children of the SEFI study, and the field research assistants and supervisors of the Vellore SEFI cohort. We also thank Mr Surendar, Mr Chandru, Ms Jackline, Mr Humayun, Mr Abbas, and Mr Selvakumar for their field support in coordinating the blood culture sampling. We are grateful for the support by study clinic team and the study physicians for the clinical support.

***Supplement sponsorship.*** This supplement is sponsored by the Christian Medical College Vellore Association.

***Financial support.*** The study was supported by the Bill & Melinda Gates Foundation (grant number OPP1159351 to G. K.).

***Potential conflicts of interest.*** All authors: No reported conflicts of interest.
