# Supplementary material for: Factors Predicting Blood Culture Positivity in Children With Enteric Fever
Source: J Infect Dis. 2021 Nov 23;224(Suppl 5):S484–93. doi: 10.1093/infdis/jiab357 (PMC8892536; doi:10.1093/infdis/jiab357)
Supplement: jiab357_suppl_Supplementary_Figure_1 [file jiab357_suppl_supplementary_figure_1.docx]

**Supplementary figure 1. Relationship between blood volume and age of the children in the SEFI cohort**

**
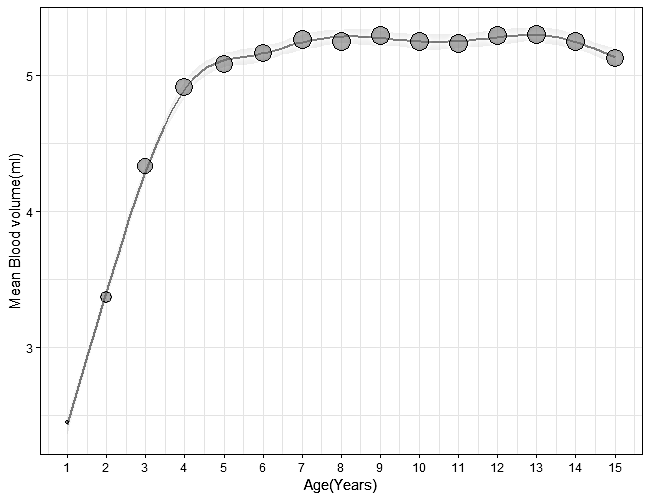
**

Mean blood volume (ml)

Age (in completed years)

Position of each circle in the graph represents the mean blood volume obtained in children during fever episodes; size of circle represents the number of fever episodes included in each age category
